# Involved/uninvolved heavy/light chain index can predict progression in transplanted multiple myeloma patients

**DOI:** 10.1038/bmt.2017.97

**Published:** 2017-06-05

**Authors:** M Espiño, A Arteche-López, S Medina, C Muñoz-Calleja, M J Blanchard, A Alegre, F J López-Jiménez, L M Villar

**Affiliations:** 1 Department of Immunology, Hospital Universitario Ramón y Cajal, Instituto Ramón y Cajal de Investigación Sanitaria, Madrid, Spain; 2 Department of Immunology, Instituto de Investigación Sanitaria Princesa, Hospital Universitario de La Princesa, Madrid, Spain; 3 Department of Hematology, Hospital Universitario Ramón y Cajal, Instituto Ramón y Cajal de Investigación Sanitaria, Madrid, Spain; 4 Department of Hematology, Instituto de Investigación Sanitaria Princesa Hospital Universitario de La Princesa, Madrid, Spain

Multiple myeloma (MM) is a neoplastic plasma-cell disorder characterized by clonal proliferation of malignant plasma cells in the bone marrow microenvironment, the presence of monoclonal protein in the blood or urine and associated organ dysfunction.^1^ In recent decades, the widespread use of autologous stem cell transplantation (ASCT) and the introduction into clinical practice of the novel agents, including proteasome inhibitors (bortezomib) and the immunomodulatory derivatives thalidomide and lenalidomide, have significantly contributed to major advances in MM therapy and prognosis.^2, 3^ Nonetheless, MM remains incurable, and the majority of patients eventually relapse after a variable period of time.

Current criteria for determining progressive disease and relapse are principally based on elevations in monoclonal protein (M-protein) or free light chains (FLC).^4^ Bone marrow plasma cell percentage and imaging techniques, such as positron emission tomography (PET) and magnetic resonance imaging are used to confirm progression, particularly in the case of MM patients without measurable M-protein.

There is a need to identify biomarkers that accurately predict relapse or progression to initiate salvage therapy that prevents serious complications such as end-organ damage.

Different parameters, such as circulating and bone marrow plasma cells or the combination of fluorodeoxyglucose-positron emission tomography/computed tomography (FDG-PET/CT) with laboratory data are suggested to predict earlier relapse in MM but they have not been validated yet.^5, 6, 7^

The aim of our study was to identify laboratory variables that could predict early MM relapse/progression in a cohort of transplanted MM patients.

We prospectively followed 44 MM-transplanted patients: 19 with IgG-kappa isotype, 11 with IgG-lambda, 9 with IgA-kappa and 5 with IgA-lambda. They were followed for 29.0±3.8 months (mean±s.e.). Serial serum samples from each of the MM patients were collected periodically after ASCT.

We considered as relapsing patients those who underwent relapse or disease progression according to International Myeloma Working Group (IMWG) consensus statement.^4^

Involved/uninvolved index (I/Ui) was calculated using the monoclonal chain (Involved) as numerator and the polyclonal chain of the same class (Uninvolved) as denominator.

The heavy/light chain (HLC) ratio (rHLC) was calculated as IgGκ/IgGλ or IgAκ/IgAλ with normal reference ranges established as 1.3–3.7 for IgG and 0.7–2.2 for IgA.^8^

To identify factors that predict disease progression in MM-transplanted patients, we studied HLC pair quantification, FLC and total Ig levels in serial serum samples collected along follow-up. Table 1 shows the results of these variables measured in basal samples (first samples obtained post ASCT) and in samples obtained at the end of follow-up or prior to relapse/progression in case of relapsing patients.

We first examined MM patients with IgG isotype. At the end of follow-up period, 11 of the 30 patients who underwent ASCT progressed, 5 showed a partial remission (PR) and 14 had an immunofixation-negative complete remission (CR).

The I/Ui was the most accurate factor to predict progression in MM-transplanted patients. In relapsing patients, this index was significantly higher in pre-relapse samples than in basal samples (2.23±0.67 vs 8.49±4.01, *P*=0.012) and anticipated progression 4.58±0.65 months in advance. Although the I/Ui remained unchanged in both CR and PR patients, we noticed that I/Ui was higher in the last group (*P*=0.002), probably due to the presence of an M component.

We next evaluated the value of the remaining variables in predicting IgG MM relapse. No differences were observed in the percentages of abnormal rHLC and rFLC during follow-up in each group of IgG MM-transplanted patients. Total IgG levels also remained stable in patients in CR along follow-up but they increased progressively in relapsing and PR patients. However, differences between basal and pre-relapse values were not significant.

Figure 1 shows representative examples of the differences between I/Ui and total IgG in the serial samples obtained from a MM patient of each group. I/Ui showed an early increase and also higher values than those of total IgG in a relapsing patient (Figure 1a). In patients in PR (Figure 1b) and CR (Figure 1c), the relative increase of I/Ui and IgG levels run in parallel along follow-up.

We next studied IgA MM patients. Out of the transplanted patients 50% relapsed and the other 50% were in CR. We observed that 71.4% of relapsing patients showed an I/Ui value above 4.0 in pre-relapse samples whereas no patient in CR showed an I/Ui value higher than 2.0 (*P*=0.02) during follow-up. No significant differences were observed in rHLC, rFLC and total IgA concentrations along follow-up in any group.

The I/Ui ratio is a quantitative marker that reflects the increase of the monoclonal (involved) chain and the decrease of the polyclonal (uninvolved) one. This index can identify MGUS patients at high risk of progression to MM,^9^ and is an independent prognostic factor in MM.^10^ We describe here that I/Ui increases earlier than total IgG levels in relapsing IgG MM-transplanted patients prior to progression. This index remained stable in patients in CR and in PR although the later ones showed higher values, thus suggesting that the presence of an M component induces a moderate immunosuppression of the uninvolved chain of the monoclonal isotype. With respect to IgA MM-transplanted patients, values of I/Ui higher than 4.0 also foresaw a progression event.

Our results show that HLC pair measurement detects early increases of M component in transplanted MM patients. Future studies will further demonstrate the role of the I/Ui ratio as a biomarker to anticipate progression in MM patients subjected to ASCT.

## Figures and Tables

**Figure 1:**
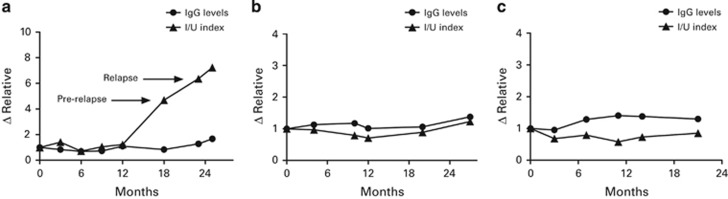
Representative example of the variations of total IgG and I/Ui along follow-up in MM transplanted patients.

**Table 1:** Results of the different variables in MM-transplanted patients during follow-up

*IgG MM (n)*			
* Relapsing patients (11)*	*Basal samples*	*Pre-relapse samples*	P
Total IgG (mg/dL, mean±s.e.)	996.3±115.4	1213.0±122.5	0.26
I/U index (mean±s.e.)	2.23±0.67	8.49±4.01	0.01
HLC ratio (abnormal/normal) (%)	7/4 (64/36)	10/1 (91/9)	0.31
FLC ratio (abnormal/normal) (%)	5/6 (45/65)	7/4 (64/36)	0.66
* Complete remission patients (14)*	*Basal samples*	*Final samples*	
Total IgG (mg/dL, mean±s.e.)	1017.0±115.4	1099.0±91.71	0.64
I/U index (mean±s.e.)	1.37±0.18	1.35±0.19	0.98
HLC ratio (abnormal/normal) (%)	9/5 (64/36)	5/9 (36/64)	0.25
FLC ratio (abnormal/normal) (%)	3/11 (21/79)	2/12 (14/86)	1.00
* Partial response patients (5)*	*Basal samples*	*Final samples*	
Total IgG (g/L, mean±s.e.)	1260.0±103.5	1530.0±155.9	0.22
I/U index (mean±s.e.)	4.43±0.55	4.55±0.35	0.84
HLC ratio (abnormal/normal)(%)	2/3 (40/60)	2/3 (40/60)	1.00
FLC ratio (abnormal/normal)(%)	2/3 (40/60)	4/1 (80/20)	0.52

*IgA MM (n)*			
* Relapsing patients (7)*	*Basal samples*	*Pre-relapse samples*	P
Total IgA (mg/dL, mean±s.e.)	267.8±75.9	474.1±94.2	0.16
I/U index (mean±s.e.)	17.7±8.7	123.0±97.6	0.45
HLC ratio (abnormal/normal) (%)	4/3	6/1	0.54
FLC ratio (abnormal/normal) (%)	3/4	6/1	0.24
* Complete remission patients (7)*	*Basal samples*	*Final samples*	
Total IgA (mg/dL, mean±s.e.)	76.9±11.9	150.9±32.5	0.13
I/U index (mean±s.e.)	1.2±0.1	1.1±0.1	0.90
HLC ratio (abnormal/normal) (%)	0/7 (0/100)	0/7 (0/100)	1
FLC ratio (abnormal/normal) (%)	1/6 (14/86)	1/6 (14/86)	1

Abbreviations: FLC= free light chains; HLC = heavy/light chain; I/U= involved/uninvolved; MM= multiple myeloma.
